# Diagnostic value of microRNA-143 in predicting in-stent restenosis for patients with lower extremity arterial occlusive disease

**DOI:** 10.1186/s40001-016-0240-y

**Published:** 2017-01-05

**Authors:** Zhi-Hai Yu, Hai-Tao Wang, Can Tu

**Affiliations:** Department of Interventional, The Affiliated Hospital of School of Medicine of Ningbo University, No. 247 Renmin Road, Jiangbei District, Ningbo, 315020 Zhejiang Province People’s Republic of China

**Keywords:** MicroRNA-143, Restenosis, Lower extremity arterial occlusive disease, Diagnostic value

## Abstract

**Purpose:**

This study was conducted to explore the diagnostic value of microRNA-143 (miRNA-143) in predicting in-stent restenosis (ISR) of lower extremity arterial occlusive disease (LEAOD).

**Methods:**

From February 2012 to March 2015, 165 patients (112 males and 53 females) with LEAOD undergoing interventional treatment were enrolled in this study. Serum miRNA-143 expression was detected using quantitative real-time polymerase chain reaction (qRT-PCR). Patients were assigned into the restenosis and non-restenosis groups according to routine surveillance postoperative angiography. A logistic regression analysis was conducted to analyze the risk factors for ISR in LEAOD patients. A receiver operating characteristic (ROC) curve was drawn to evaluate the diagnostic value of miRNA-143 in predicting ISR for LEAOD patients.

**Results:**

There were 74 and 91 patients in the restenosis and non-restenosis groups, respectively. Before the treatment, there were significant differences in history of diabetes, smoking status, blood sugar level (BSL) at admission, low-density lipoprotein cholesterol (LDL-C) level, and stent diameter between the restenosis and non-restenosis groups (all *P* < 0.05). Serum miRNA-143 expression was lower in the restenosis group than in the non-restenosis group (*P* < 0.05). Serum miRNA-143 expression in the restenosis group was correlated with smoking status, history of diabetes, BSL, and LDL-C (all *P* < 0.05). Logistic regression analysis demonstrated that miRNA-143, LDL-C, and smoking status were correlated with the postoperative ISR (all *P* < 0.05). ROC curve analysis revealed that the area under the curve (AUC) of miRNA-143 in predicting ISR for LEAOD patients was 0.866.

**Conclusion:**

Our results indicate that miRNA-143 can be a promising tool for predicting the ISR in LEAOD patients.

## Background

Lower extremity arterial occlusive disease (LEAOD), derived from atherosclerosis, is a vascular disease with a high incidence of coronary artery disease and even limb loss worldwide [1]. Currently, endovascular interventional treatment, with advanced techniques and devices, has been widely used in the treatment of artery occlusive disease [2, 3]. Despite endovascular interventional treatment’s high success rate and repeatability, one of its implications is in-stent restenosis (ISR), which results from a reduced vessel patency in the wake of vascular endothelial injury and intimal hyperplasia followed by thrombosis. ISR can influence the prognosis of patients [4, 5]. A previous study has suggested that intimal hyperplasia is implicated in the pathogenesis of restenosis, which constitutes the major cause of treatment failure [6]. Luminal narrowing and intimal hyperplasia are correlated with the proliferation of vascular smooth muscle cells (VSMCs), which make it possible to prevent ISR by inhibiting the highly proliferating VSMCs [7]. Therefore, it is important to explore how molecular biology and genetics affect the migration and proliferation of VSMCs, which may lead to an effective method for the prediction and diagnosis of ISR.

MicroRNAs (miRNAs) are a novel class of endogenous, non-protein coding, small RNA molecules that can modulate hundreds of genes [8]. Their altered levels have been reported in patients with various diseases, such as heart failure and coronary artery disease [9]. Found in a wide range of human diseases, miRNAs have been identified as a novel biomarker for various physiological and pathological conditions [10]. It has been discovered that miRNA-143, lying on human chromosome 5 (1.7 kb), and highly expressive in VSMCs, plays a crucial part in the migration and proliferation of VSMCs [11]. Importantly, the expression of miRNA-143 has been reported to vary with the oscillation of VSMCs between differentiated and proliferative phenotypes [12]. Although several previous studies have been conducted to probe the expression of miRNAs in LEAOD [1, 13], few have reported details of the correlation between miRNA-143 and ISR in LEAOD. This study was conducted to provide a better solution to intervening in LEAOD with further insight into the molecule’s diagnostic value in predicting ISR based on its expression.

## Methods

### Ethics statement

This study was approved by the Ethical Committee of the Affiliated Hospital of School of Medicine of Ningbo University, and all participants have signed the informed consent.

### Subjects

From February 2012 to March 2015, a total of 165 patients (112 males and 53 females, mean age: 65.0 ± 8.3 years) aged from 45 to 78 years old with LEAOD who underwent treatment in the Department of Vascular Surgery of the Affiliated Hospital of School of Medicine of Ningbo University were enrolled in this study. All subjects were diagnosed with LEAOD at different levels after undergoing computed tomography angiography (CTA). The inclusion criteria were as follows: weakened or lack of arterial pulse, nutritional disorders, and paleness in the lower extremities. Risk factors such as hyperglycemia, hyperlipidemia, hypertension, age, and smoking status were taken into consideration. Exclusion criteria were as follows: (1) patients with an implanted stent who refused to undergo a CTA review; (2) patients who did not take anti-platelet aggregation drugs; (3) patients who had ISR in two or more locations and received revascularization therapy within 1 month after the diagnosis of ISR; (4) patients with severe left ventricular dysfunction whose ejection fraction was below 40%; (5) patients with combined incomplete function of important organs, such as liver dysfunction (various pathogenic factors cause serious damage to the liver parenchymal cells and Kupffer cells), kidney dysfunction (serious glomerular damage, body disorders in the excretion of metabolic waste and water electrolyte and acid–base regulation balance), lung dysfunction (respiratory failure), brain dysfunction (brain injury and concussion), infection (bacteria, viruses, and parasites), poisoning (organic phosphorus poisoning and poisonous gas), cardiac and cerebral vascular diseases (cerebral hemorrhage, cerebral thrombosis and hypertension), brain aging, brain tumors, and other dysfunction of water, electrolyte, and pH balance, osmotic pressure, and other internal environmental factors; (6) patients who had connective tissue diseases, autoimmune diseases, or malignancy; and (7) patients who had a history of acute myocardial infarction.

### Interventional treatment

An appropriate interventional treatment was chosen based on preoperative CTA imaging. The ipsilateral femoral artery was the top priority if stenosis or occlusion occurred in the middle or distal third segment of the superficial femoral artery or in the popliteal artery. When stenosis or occlusion occurred in the common femoral artery, proximal third segment of the superficial femoral artery, or the iliac artery, the patient required an intracavitary therapy (including catheter directed thrombolysis (CDT), pharmaco mechanical CDT (PCDT), percutaneous aspiration (PAT), protective inserting Greenfield caval filter, and balloon dilation combined with stent implantation) [14]. A retrograde puncture of the lateral femoral artery to conduct the intracavitary therapy was made with “crosses sheath.” If the bilateral femoral arteries were not in proper condition to be punctured in this way, the puncture was made through the brachial artery. After the end of the puncture, patients who underwent local anesthesia were implanted with a sheathing canal, through which angiography of the targeted arteries was carried out to select the therapeutic regimen. Percutaneous transluminal angioplasty (PTA) was employed for patients whose vascular stenosis was shorter than 3 cm while an endovascular stent (ES) was used for patients whose residual stenosis accounted for more than 30% after PTA. For the patients with arterial stenosis longer than 10 cm or with total occlusion, rotational atherectomy was applied. Under those conditions, different types of guidewire were chosen based on different approaches and forming methods. The ev3 NanoCross and Bantam were selected for PTA microballoon, and the self-expandable metallic stents (ev3 ProtegeEverFlex and BARD LifeStent XL) were adopted as the stent.

### Sample collection

A total of 10 mL of venous blood was obtained from all patients (anticoagulant tubes containing sodium citrate). Plasma was obtained within 4 h before it was centrifuged in accordance with the standard Ficoll density gradient centrifugation. Centrifugal conditions were as follows: 1800*g* at room temperature for 10 min and let sit at room temperature. Plasma was bottled into 1-mL nuclease Eppendorf (EP) tubes and refrigerated in an ultra-low-temperature refrigerator at −80 °C. Five μL of the RNA sample was taken out for RNA extraction using the miRNeasy Mini Kit (Applied Biosystems Company, CA, USA) in accordance with the instructions. According to the instructions of the Tiangen miRcute miRNA cDNA first strand synthesis reagents kit (Fermentas, Thermo Fisher Scientific, Waltham, Massachusetts, USA), miRNA was first modified by adding Poly (A) to the end of the miRNA3 and then centrifuged for a short time before its reaction, followed by a brief centrifugation of reverse transcription with the universal Oligo (DT)-Universal Tag primer. Next, it reacted for 60 min at 37 °C, followed by the generation of the first strand of cDNA, the counterpart of miRNA, which was then placed under −20 °C and let sit.

### Quantitative real-time polymerase chain reaction (qRT-PCR)

Serum miRNA-143 expression was measured in the plasma of each patient using qRT-PCR. U6 was employed as an internal control. Primers were synthesized by the Shanghai Invitrogen company (Shanghai, China). Primer sequences are presented in Table 1. The testing process was carried out in accordance with the instructions of Taqman miRNA assays, and the reaction was conducted with the Prism Sequence Detection System ABI HT 7900 PCR instrument. The conditions for PCR circulation involved the following: 40 cycles of pre-denaturation at 95 °C for 20 s, degeneration at 95 °C for 10 s, annealing at 60 °C for 20 s, and extension at 70 °C for 20 s. This procedure was repeated three times for each sample with a total reaction volume of 20 μL. The relative expression was calculated using 2^−ΔΔCt^ (∆∆Ct = ∆Ct (test specimen)—∆Ct (authentic specimen), ∆Ct (test specimen) = Ct (test specimen, target gene)—Ct (test specimen, reference gene), ∆Ct (authentic specimen) = Ct (authentic specimen, target gene)—Ct (authentic specimen, reference gene), 2^−∆∆Ct^ = 2^−(1.2)^ = 2.30).

**Table 1:** Primer sequence of quantitative real-time polymerase chain reaction (qRT-PCR)

Primer	Sequence (5′–3′)	Length (bp)
U6	Forward: GTTTTGTAGTTTTTGGAGTTAGTGTTGTGT	135
	Reverse: CTCAACCTACAATCAAAAACAACACAAACA	
miR-143	Forward: TGTAGTTTTCGGAGTTAGTGTCGCGC	556
	Reverse: CCTACGATCGAAAACGACGCGAACG	

### Follow-up

Discharged patients were followed up to further understand their condition change and recovery situation. During their return visit, their symptoms of claudication and arterial pulse were examined. Postoperative CAT scanning was conducted, and the ankle-brachial index (ABI) was measured. Criteria for effective treatment include the following: (1) stenosis in angiography vessel lumen less than 30%; (2) no obvious artery dissection or surgery-related complications; (3) resolution or improvement in claudication, rest pain, and other symptoms in the lower extremities as well as ulcer healing. If these criteria were not met, the treatment was regarded as invalid. Based on their recovery level, patients were assigned into the ISR group (*n* = 74, 52 males and 22 females, mean age: 65.4 ± 8.6 years) and the non-ISR group (*n* = 91, 60 males and 31 females, mean age: 64.7 ± 8.0 years).

### Statistical analysis

SPSS21.0 statistical software was used for data analysis. Measurement data were presented as mean ± standard deviation (SD). The *t* test was used for the comparison between the two groups. Count data were expressed as a percentage or rate. Comparisons between the two groups were analyzed using the Chi-square test. Logistic regression analysis was performed to analyze the risk factors for postoperative ISR of LEAOD patients. A receiver operating characteristic (ROC) curve was drawn using statistical software to evaluate the diagnostic value of miRNA-143 in predicting ISR in LEAOD patients. *P* < 0.05 was considered statistically significant.

## Results

### Comparison of clinical features of LEAOD patients between the restenosis and non-restenosis groups

A total 165 patients who had undergone interventional treatment of LEAOD were followed up and assigned into the restenosis group (*n* = 74) or the non-restenosis group (*n* = 91). The baseline characteristics of the two groups are compared in Table 2. There were significant differences in multiple indices before treatment, including history of diabetes, smoking status, blood sugar level (BSL) at admission, low-density lipoprotein cholesterol (LDL-C) level, stent diameter, etc. (all *P* < 0.05). However, there were no significant differences in age, gender, hypertension, or hyperlipidemia between the two groups (all *P* > 0.05).

**Table 2:** Comparison of clinical features of patients in the restenosis and non-restenosis groups

Feature	Restenosis group (*n* = 74)	Non-restenosis group (*n* = 91)	*P*
Age (years)	65.4 ± 8.6	64.7 ± 8.0	0.583
Male, n (%)	52 (70.2)	60 (65.9)	0.553
BMI (kg/m^2^)	24.8 ± 3.6	24.1 ± 3.2	0.184
Hypertension, n (%)	19 (25.7)	22 (24.2)	0.825
Hyperlipidemia, n (%)	15 (20.3)	17 (18.7)	0.797
Renal dysfunction, n (%)	10 (13.5)	11 (12.1)	0.785
Diabetes, n (%)	21 (28.4)	6 (6.6)	<0.001
Smoking, n (%)	59 (79.7)	27 (29.7)	<0.001
Drinking, n (%)	33 (44.6)	40 (44.0)	0.935
SBP (mmHg)	137.8 ± 22.3	133.6 ± 21.5	0.221
DBP (mmHg)	73.4 ± 9.8	72.6 ± 7.7	0.558
HDL-C (mmol/L)	1.1 ± 0.3	1.2 ± 0.3	0.177
LDL-C (mmol/L)	3.0 ± 0.5	2.5 ± 0.3	<0.001
Blood sugar, (mmol/L)	6.3 ± 2.0	5.4 ± 1.8	<0.001
Stent mean diameter (mm)	7.4 ± 1.0	6.6 ± 1.0	<0.001

*BMI* body mass index; *SBP* systolic blood pressure; *DBP* diastolic blood pressure; *HDL-C* high-density lipoprotein cholesterol; *LDL-C* low-density lipoprotein cholesterol

### Comparison of serum miRNA-143 expression between the restenosis and non-restenosis groups

**Fig. 1:**
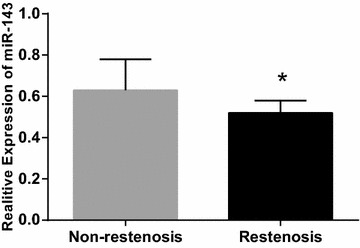
Comparison of microRNA-143 expression between the restenosis and non-restenosis groups. *Compared with the non-restenosis group, *P* < 0.05

There were significant differences in serum miRNA-143 expression between the two groups. Compared with the non-restenosis group, serum miRNA-143 expression in the restenosis group was decreased (*P* < 0.05) (Fig. 1).

### Correlation of serum miRNA-143 expression with clinicopathological features between the restenosis and non-restenosis groups

As presented in Table 3, the serum miRNA-143 expression in the restenosis group was correlated with smoking status, history of diabetes, BSL, and LDL-C (all *P* < 0.05), but it had no correlation with age or hyperlipidemia (both *P* > 0.05). In the non-restenosis group, serum miRNA-143 expression had no correlation with age, smoking status, history of diabetes, BSL, LDL-C, or hyperlipidemia (all *P* > 0.05).

**Table 3:** The relationships between microRNA-143 expression and clinical features of patients in the restenosis and non-restenosis groups

Feature	Restenosis group (n = 74)				Non-restenosis group (n = 91)			
	*N*	Relative expression	*t*	*P*	*N*	Relative expression	*t*	*P*
Age (years)								
<60	40	0.53 ± 0.07	1.39	0.169	49	0.64 ± 0.14	0.64	0.526
≥60	34	0.51 ± 0.05			42	0.62 ± 0.16		
Smoking								
Yes	59	0.50 ± 0.04	7.75	<0.001	27	0.62 ± 0.14	0.28	0.779
No	15	0.60 ± 0.06			64	0.63 ± 0.16		
Diabetes								
Yes	21	0.48 ± 0.04	3.51	0.001	6	0.62 ± 0.20	0.16	0.878
No	53	0.53 ± 0.06			85	0.63 ± 0.15		
Blood sugar								
High	14	0.45 ± 0.04	6.27	<0.001	19	0.60 ± 0.12	1.11	0.313
Low	60	0.54 ± 0.05			72	0.64 ± 0.16		
LDL-C								
>2.6 mmol/L	35	0.49 ± 0.06	5.11	<0.001	20	0.61 ± 0.12	0.78	0.439
≤2.6 mmol/L	39	0.55 ± 0.04			71	0.64 ± 0.1		
Hyperlipidemia								
Yes	15	0.51 ± 0.04	0.61	0.544	17	0.64 ± 0.15	0.74	0.459
No	59	0.52 ± 0.06			74	0.63 ± 015		

*LDL-C* low-density lipoprotein cholesterol

### Logistic regression analysis of risk factors for postoperative ISR in LEAOD patients

As shown in Table 4, serum miRNA-143 expression, LDL-C, and smoking status were correlated with postoperative ISR (all *P* < 0.05). Both smoking status and LDL-C level were risk factors for ISR in LEAOD patients.

**Table 4:** Logistic regression analysis of risk factors for postoperative restenosis in patients with lower extremity arterial occlusive disease

Factor	OR	95% CI	*P*	Partial regression coefficient	*χ* ^2^	Standard error
miR-143	0.001	0.000–0.077	0.001	−6.515	10.475	2.013
Blood sugar	0.905	0.708–1.157	0.427	−0.1	0.63	0.125
LDL-C	9.953	2.629–37.678	0.001	2.298	11.446	0.679
Diabetes	1.31	0.368–4.670	0.677	0.27	0.174	0.648
Smoking	5.12	2.069–12.673	<0.001	1.633	12.476	0.462

*miR-143* microRNA-143; *LDL-C* low-density lipoprotein cholesterol

### ROC curve analysis for the diagnostic value of miRNA-143 in predicting ISR in LEAOD patients

**Fig. 2:**
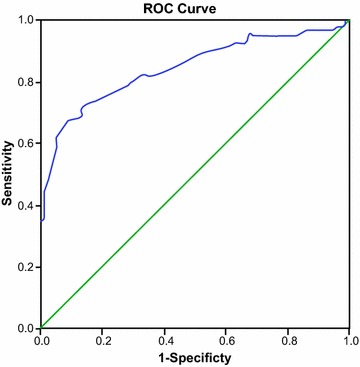
ROC curve analysis for the diagnostic value of microRNA-143 in predicting ISR in LEAOD patients. ROC curve, receiver operating characteristic curve; ISR, in-stent restenosis; LEAOD, lower extremity arterial occlusive disease

The area under the curve (AUC) was 0.866, which suggested that miRNA-143 is a promising tool for predicting ISR in LEAOD patients (Fig. 2). Furthermore, the sensitivity and specificity of miRNA-143 in predicting ISR in LEAOD patients were 83.7 and 82.6%, respectively.

## Discussion

In recent years, endovascular interventional treatment has been an important treatment for the occlusive or stenosis diseases, especially LEAOD, of atherosclerosis [15], but postoperative restenosis remains as a big challenge for the treatment [1]. Although some studies have attempted to prevent ISR, we still do not have an optimal treatment [16]. Thus, finding an independent predictor of restenosis is of great significance for early management and prevention of ISR.

This study found that serum miRNA-143 expression in the restenosis group was significantly decreased when compared with the non-restenosis group. The logistic regression analysis also demonstrated that serum miRNA-143 expression was inversely related to the postoperative ISR rate in LEAOD patients. The major pathogenesis of posttreatment restenosis was the migration and proliferation of VSMCs. VSMCs can be stimulated by endothelial injury so that the medial and adventitial layers migrate to the intimal layer. Then, the neointima is generated and can lead to restenosis [17]. MiRNAs are known as vital regulators of many cellular events, and as a member of the miRNA family, miRNA-143 was reported to play a role in modulating VSMC phenotypes [18]. The serum response factor (SRF) is a transcription factor that plays a vital role in the regulation of VSMCs. It plays the role of a common docking site for both myogenic coactivators and myogenic corepressors in VSMC phenotypic switching. Thus, as the transcription factor of miRNA-143, Ets-like transcription factor-1 (ElK-1) combines with the SRF to repress VSMC growth [12]. In addition, angiotensin-converting enzyme (ACE-1) is one of the targets of miRNA-143. Also, ACE inhibitors have the potential to reverse vascular dysfunction [19]. It was found that serum miRNA-143 expression was decreased in states of atherosclerosis [20]. It was also discovered that serum miRNA-143 expression was decreased in the medial and intimal layers of the coronary artery during the development of restenosis [21]. Patients in the ISR group had a significantly lower serum miRNA-143 expression than in the non-restenosis group. Also, miRNA-143 was found to have a higher sensitivity and specificity for ISR diagnosis. Thus, miRNA-143 can be regarded as a biomarker for ISR [22]. This study also found that serum miRNA-143 expression was decreased in the restenosis group compared with the non-restenosis group, suggesting that miRNA-143 has a good predictive ability for postoperative restenosis. Therefore, it can be concluded that miRNA-143 can be involved in ISR development by regulating VSMCs, and its expression can be used to predict postoperative restenosis.

In the logistic regression analysis of relevant factors of restenosis, it was found that LDL-C and smoking status were the risk factors for restenosis. Because serum miRNA-143 expression levels are correlated with ISR occurrence, miRNA-143 potentially participates in the process of ISR development [22]. LDL-C is involved in the development of atherosclerosis and is an important risk factor for coronary artery disease (CAD). The regulation of LDL-C can contribute to reductions in ISR rates [23]. In a previous study, smoking status was also predicted to be one of the risks factors of ISR development [24]. Our study also demonstrates the relevance of miRNA-143, LDL-C, and smoking status to postoperative restenosis by logistic regression analysis. Thus, these factors can be considered risk factors for restenosis.

In conclusion, RNA-143 expression plays an important role in predicting restenosis in LEAOD patients, and can offer a theoretical basis for the treatment of LEAOD so that restenosis can be controlled by the regulation of miR-143 in VSMCs. However, more research needs to be done to explore the down-regulation mechanism of miNAR-143 in restenosis development to determine the most effective way to reduce the rate of restenosis by regulating miRNA-143.

## Abbreviations

miRNA-143: microRNA-143
ISR: in-stent restenosis
LEAOD: lower extremity arterial occlusive disease
ROC: receiver operating characteristic
LDL-C: low-density lipoprotein cholesterol
AUC: area under the curve
VSMCs: vascular smooth muscle cells
CTA: computed tomography angiography
PTA: percutaneous transluminal angioplasty
ES: endovascular stent
EP: eppendorf
qRT-PCR: quantitative real-time polymerase chain reaction
ABI: ankle-brachial index
SD: standard deviation
SRF: serum response factor
ElK-1: Ets-like transcription factor-1
CAD: coronary artery disease
